# Investigating the effectiveness of intraoperative rapid parathyroid hormone assay in parathyroidectomy surgery in patients with secondary hyperparathyroidism

**DOI:** 10.1186/s12902-023-01378-3

**Published:** 2023-05-25

**Authors:** Shirzad Nasiri, Seyed Mostafa Meshkati Yazd, Alireza Heshmati, Abnoos Mokhtari Ardekani, Masoud Najafi, Reza Shahriarirad

**Affiliations:** 1 Department of Surgery, Tehran University of Medical Sciences, Tehran, Iran; 2 Endocrinology and Metabolism Research Center, Institute of Basic and Clinical Physiology Science, & Physiology Research Center, Kerman University of Medical Sciences, Kerman, Iran; 3 School of Medicine, Shiraz University of Medical Sciences, Shiraz, Iran; 4 Thoracic and Vascular Surgery Research Center, Shiraz University of Medical Science, Shiraz, Iran

**Keywords:** Secondary hyperparathyroidism, Parathyroidectomy, Intraoperative Rapid PTH

## Abstract

**Introduction:**

The use of Rapid Intraoperative parathyroid hormone (Io-PTH) assay during surgery in the management of parathyroid tissue in cases of primary hyperparathyroidism has been proven to be effective, while its utilization in secondary hyperparathyroidism (SHPT) has been rarely reported. In the present study, we aim to demonstrate the application of rapid Io-PTH assay in patients with SHPT following chronic kidney disease undergoing parathyroidectomy surgery.

**Method:**

In this prospective study, five blood samples were taken from patients undergoing parathyroidectomy and upper thymectomy. Among the samples, two were pre-excision, including prior to the first incision, after exploration, and before parathyroids resection. Two additional samples were taken 10 and 20 min after the excision of the parathyroid glands. Another sample was collected twenty-four hours after the operation. Serum Calcium levels and PTH levels were evaluated and analyzed.

**Results:**

We successfully managed SHPT in all 36 patients in our study. The patients included 24 males (66.7%) with a mean age of 49.97 ± 14.92. The mean PTH decreased significantly at 10 min, 20 min, one day, and six months after surgery (*P* < 0.001). The highest reduction occurred 10 min after removal of the parathyroid glands so the mean PTH compared to time zero was reduced from 1737 to 439, and in 100% of cases, more than 50% reduction was seen in PTH.

**Conclusion:**

A 60% or more reduction in PTH Rapid at 10 min after parathyroidectomy has an accuracy of 94.4% and a positive predictive value of 100%. Thus, if the PTH level does not decrease by more than 60% at 10 min or more than 80% at 20 min, tissue exploration is continued with the aim of finding the ectopic parathyroid gland.

## Highlights

- A 60% or more reduction in rapid Io-PTH at 10 minutes after parathyroidectomy has an accuracy of 94.4% and a positive predictive value of 100%.
- If the PTH level does not decrease by more than 60% at 10 minutes or more than 80% at 20 minutes, accurate exploration should be performed again.
- If the remaining glands have not yet been identified, postoperative localization studies should be performed.

## Introduction

Secondary hyperparathyroidism (SHPT) is one of the most common complications of chronic kidney disease (CKD), in which the homeostasis of calcium and phosphorus is impaired [1]. This complication is associated with significant morbidity and mortality in patients requiring renal replacement therapy [2]. However, SHPT often needs proper treatment as it does not resolve spontaneously with kidney transplantation [3]. Management can be achieved by pharmacologic agents such as calcimimetics, calcium supplements, phosphor binders, and Calcitriol, as well as surgical interventions like total and subtotal parathyroidectomy [4], which are usually indicated when the patient does not respond to medical therapy [5].

Attempts have been made to avoid surgical and management complications. Such interventions might not be successful and present as persistent hyperparathyroidism due to ectopic and additional parathyroid glands [6]. On the other hand, unnecessary exploration of the neck region can cause multiple complications as damage to recurrent laryngeal nerves [7]. In this regard, real-time monitoring of the decline in plasma parathyroid hormone (PTH) levels, such as Intraoperative parathyroid hormone (Io-PTH) measurement, can result in fewer complications of parathyroidectomy by proving further exploration and surgical resections unnecessary [8, 9]. In this study, we aimed to assess the effectiveness of Io-PTH measurement in the success of total and subtotal parathyroidectomy SHPT surgeries.

## Methods and materials

### Study design

This study followed a descriptive protocol on a cohort of patients undergoing total or subtotal parathyroidectomy due to secondary hyperparathyroidism. The usefulness of intraoperative rapid PTH for decision-making regarding the continuation or termination of surgery was assessed in the patients, and they were followed for about one year after the intervention. The results of their lab studies were used as an indicator of treatment success, and they were evaluated for any significant adverse effect.

### Participants

The inclusion criteria of the study were patients between 15 and 75 years old with secondary hyperparathyroidism following CKD, who were referred from the nephrology and endocrinology clinics to our endocrine surgery clinic of Shariati hospital for total (in case of the patient not being a candidate for kidney transplantation) and subtotal (in case of the patient being a candidate for kidney transplantation) parathyroidectomy. Patients with a previous history of neck surgery or post-transplant SHPT were excluded for our study. The recruitment of the patients lasted from March 2019 to March 2020, and the follow-up continued until 2021. Based on the surgical indications for secondary hyperparathyroidism modified by the Japanese society for dialysis therapy guideline, persistent intact PTH > 500 is considered for surgery [10].

### Variables and data sources

After explaining the study design and obtaining a written consent form from the patients, they were enrolled in the study. Five blood samples were taken from the patients. The first one was drawn after anesthesia induction and before the first incision of the skin. Then the second one was taken after a full exploration of parathyroid glands and before parathyroid resection. The two samples were considered as pre-excision samples for PTH assessment. In patients undergoing subtotal parathyroidectomy, approximately 50 mg of the most normal parathyroid gland was preserved, and the rest were excised completely. Then, the surgery was accomplished with upper thymectomy. Intraoperative neural monitoring was also performed to avoid nerve damage. 10 and 20 min after the excision of the parathyroid glands, two blood samples were taken from the patients. In cases undergoing total parathyroidectomy and upper thymectomy, all parathyroid glands with upper part of thymus were excised completely, and then blood samples were drawn 10 and 20 min after the excision. We waited for the frozen section and lab results of the patients. No tissue manipulation was performed during the 10 to 20 waiting period. When frozen sections confirmed the excision of parathyroid glands, 50–80 mg of apparently normal parathyroid tissue were auto-transplanted to the patients' subcutaneous pocket in the infra-clavicular area, as previously reported [11]. If lab results indicated a more than 50% decline in serum PTH in post-excision samples compared to pre-excision samples, the intervention was assumed complete. If this endpoint was not achieved while the frozen section evaluation also doesn’t confirm the total resection of four parathyroid glands, the surgery was assumed incomplete, and further search for ectopic and residual parathyroid gland(s) will be carried out, including exploration of the most common sites of ectopic parathyroid glands in patients with persistent HPT such as the paraesophageal and intrathyroidal regions. Twenty-four hours after the operation, a fifth blood sample was obtained from the patients to assess PTH levels. All patients receive 10 gr calcium gluconate daily intravenously and calcium carbonate and Rocaltrol. Serum Calcium levels were checked and corrected based on albumin levels every six hours for the patient to assess for life-threatening hypocalcemia, and proper therapeutic measures were taken. Six months after the surgery, the patients were evaluated with serum PTH and Calcium levels and physical examination. In cases of hypocalcemia, 1gr of intravenous calcium, Rocatrol and 6gr of oral calcium was administered similar to previous studies [12]. Finally, all patients with calcium above 8 and only with oral calcium were discharged and were followed up in regular visits.

### Study size and statistical analysis

The data were entered into IBM SPSS version 26.0. the qualitative data were reported as frequency and percentages, and the quantitative variables were reported as mean and standard deviation. Repeated Analysis of Variances (Repeated ANOVA) was used to compare the results of serum PTH in different samples. P-values less than 0.05 were considered statistically significant. Based on the type of study and considering the average of 15 min after surgery as the most appropriate time for PTH measurement and considering a 1% accuracy, the minimum sample size for the study was 36 participants.

## Results

All endpoints were met among the 36 patients who entered the study. The mean age of the participants was 49.97 ± 14.92 years old, and 24 (66.7%) were male. The most common presenting symptoms of the patients were myalgia (50%), weakness, and pruritus. All patients required hemodialysis prior to the surgery. Table 1 summarizes the demographic information of the patients.

**Table 1:** Baseline demographic and clinical information of the patients undergoing parathyroidectomy

**Variable**	**Frequency (%)**; *N* = 36
**Age (years)**	
15–30	3 (8.3)
30–45	13 (36.1)
45–60	7 (19.4)
60–75	13 (36.1)
**Sex**	
Male	24 (66.7)
Female	12 (33.3)
**Chief complaint**	
Myalgia	18 (50.0)
Flushing and sweating	2 (5.6)
Pruritus	5 (13.9)
Hypercalcemia	2 (5.6)
Weakness	9 (25.0)
**Number of parathyroid glands in sestamibi scan**	
1	8 (22.2)
2	10 (27.8)
3	1 (2.8)
4	15 (44.4)
**Number of parathyroid glands in sonography**	
1	1 (2.8)
2	11 (30.6)
3	10 (27.8)
4	14 (38.9)
**Number of parathyroid glands in surgery**	
4	34 (94.4)
5	2 (5.6)
**Type of Thyroidectomy**	
Subtotal	2 (5.6)
Total	34 (94.4)

The correlations between sonography and sestamibi scan findings with surgical findings were weak, in that neither sonography nor sestamibi scan could find functional parathyroid tissues.

Serum PTH levels of the patients at different times were compared, which showed a significant decrease in PTH levels 10 min and 20 min post-excision, as well as 24 h and six months after the surgery. (*P* < 0.001) The most significant decline in PTH level was seen within the first ten minutes after the operation (from 1737 to 439 pg/mL or 74.7%), which then declined to 260 pg/mL after 20 min (85.0% decline). All of the patients experienced a more than 50% decline in their PTH levels during this period. Table 2 summarizes the PTH levels of the patients prior to operation, during surgery, and in the follow-up period. According to the results of ANOVA of rapid Io-PTH, PTH measurement 10 min after parathyroidectomy due to strong correlation with PTH evaluation, at 20 min, had a high predictive power for surgical success. After parathyroidectomy, a 60% or more reduction in rapid Io-PTH at 10 min has an accuracy of 94.4% (34 out of 36 patients) and a positive predictive value of 100%.

**Table 2:** Comparison of parathyroid hormone (PTH) levels of the patients at different times

**Check Points**	**Mean ± SD (pg/mL)**	***P*-value**^a^	**Time**	***P*-value**^a^
**T1: Baseline PTH**	1952.39 ± 484.37	1 vs. 2:** 0.052**	Time 2	Time 2 vs. 3: < **0.001**
**T2: Operation time PTH**	1739.500 ± 520.32			
**T3: 10 min PTH**	437.344 ± 190.27	3 vs. 4:** < 0.001**	Time 3	
**T4: 20 min PTH**	260.874 ± 142.84			Time 3 vs. 4: < **0.001**
**T5: 24-h PTH**	27.936 ± 25.66	5 vs. 6:** < 0.001**	Time 4	
**T6: 6-month PTH**	32.133 ± 12.707			

*SD* Standard deviation

^a^Repeated measure analysis of variance (ANOVA) was used to assess the significance

Two patients showed PTH reduction of less than 60% after 10 min and less than 80% at 20 min after removal of the four glands, in which we detected an extra parathyroid (ectopic) gland in the paraesophageal area which was confirmed with frozen section. However, the final results of PTH measurement (at one day and six months after surgery) in these two patients had returned to normal (below 50).

**Fig. 1:**
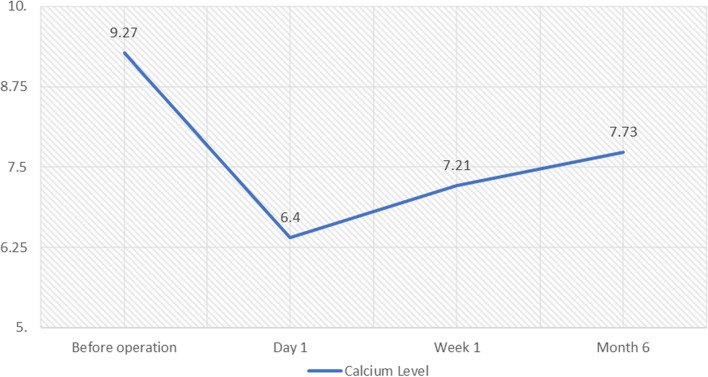
Calcium levels after parathyroidectomy

Serum Calcium levels of the patients were evaluated prior to the surgery and one day, seven days, and six months after the operation. The mean results were 9.267 ± 1.92, 6.397 ± 0.76, 7.208 ± 0.94, and 7.728 ± 0.73 mg/dL, respectively (Fig. 1). The Calcium levels decreased significantly, and none of the patients developed signs and symptoms of hypercalcemia in the follow-up period; however, all patients were advised to consume oral calcium supplements to keep their calcium levels within normal range.

It is worth mentioning that no other complications were observed among the patients, including recurrent nerve injury or postoperative hemorrhage.

## Discussion

Io-PTH measurement's effectiveness in managing the parathyroid gland in the context of primary hyperparathyroidism, both adenoma and hyperplasia, have been reported [13, 14]. Direct unilateral neck exploration based on preoperative evaluation and localization, along with Io-PTH assessment, can be beneficial in decreasing surgery time, costs, and morbidity. However, bilateral routine exploration in Multiple endocrine neoplasia (MEN) syndromes and SHPT are still a matter of debate [15–17]. The application of Io-PTH monitoring with a diagnostic accuracy of 93–98% based on other studies is a good predictor of complete resection and success in abnormal parathyroid resection [18]. Therefore, our study aimed to evaluate the effectiveness of rapid Io-PTH in the surgical management of SHPT patients.

Since total or subtotal parathyroidectomy is required for successful treatment, a 50% reduction in PTH is likely insufficient to estimate the appropriate treatment response in SHPT [15–17, 19]. Using rapid Io-PTH measurement and evaluation, the mean PTH reduction in this study was 74.7% at 10 min and 85% at 20 min. Patients had low PTH levels and significant recovery during the follow-up period. Only two patients showed PTH reduction of less than 60% after 10 min and less than 80% at 20 min after removal of the four glands and upper part of thymus, in which we had detected an extra parathyroid (ectopic) gland in the paraesophageal area. However, their PTH levels converted to normal during follow-ups due to ectopic parathyroid removal during re-exploration.

Observing that PTH levels during surgery are greatly affected by surgical manipulations is consistent with previously published results [19, 20]. However, as in previous studies, our results revealed that surgery significantly reduced PTH levels (about 74% at 10 min and 85% at 20 min after surgery) [17]. According to the analysis of repeated measurements performed on Rapid Io-PTH, it was shown that PTH measurement 10 min after parathyroidectomy due to strong correlation with PTH evaluation, at 20 min, had a high predictive power for surgical success. After parathyroidectomy, a 60% or more reduction in Rapid Io-PTH at 10 min has an accuracy of 94.4% (34 out of 36 patients) and a positive predictive value of 100%. This result is in line with other limited studies conducted in this area. For example, in a study conducted by Enrico Di Stacio et al. on 145 primary hyperparathyroidism patients who underwent parathyroidectomy using Minimally Invasive and Video-assisted methods, the time of 20 min was introduced as the endpoint for determining the success of parathyroidectomy using Io-PTH [21]. However, in a study conducted by Vulpio et al. on 42 SHPT patients who underwent parathyroidectomy, the measurement of serum PTH levels 30 min after parathyroid excision (post-excision) had a diagnostic accuracy of 93% and positive predictive value of 100% in predicting SHPT persistence after surgery [22]. Based on our analysis, PTH evaluation at 10 min after parathyroidectomy (with a reduction rate of more than 60% at the PTH level) has the same accuracy as at the 20 min. Thus, it can save 10 min and also avoid unnecessary tissue manipulation.

Among the cases in our study, ioPTH changed the management in 2 out of 36 (5.6%) of the patients. Persistent SHPT has been reported to range from 4% to up to 30% even in the hands of experienced surgeons in various references [19, 23–26]. The recurrence can be due to ectopic or supernumerary glands in the neck or mediastinum. In this regard, imaging modalitis such as ultrasound and sestamibi scans has also fallen short in the detection of hyperplastic and supernumerary glands [27–33]. In addition, bilateral exploration and reoperation in SHPT patients can increase the risk of complications including injury to the recurrent laryngeal nerve. Therefore, we believe that the efficacy of ioPTH measurement in SHPT is vital in order to reduce the possibility of reoperation due to supernumerary glands.

Total thyroidectomy was performed in two of our patients, while the remaining underwent subtotal thyroidectomy. But based on the results of our center as well as the systematic study and meta-analysis of Juan Chen et al., the long-term results of both methods have been reported similar in terms of PTH level [23]. When the parathyroid glands are manipulated, subsequent hematoma and vasoconstriction may result in abnormal function of PTH secretion. In this regard, subtotal or total parathyroid surgery does not affect the ioPTH secretion. Also, based on similar studies, measuring ioPTH has been shown to be effective in subtotal SHPT surgery, and high ioPTH may probably be due to supernumerary glands that have been missed [34]. However, further studies in this regard are warranted to provide further information and details regarding the most optimal method of surgery and management of SHPT.

Io-PTH monitoring shows that the reduction in rapid PTH after parathyroidectomy for SHPT is approximately similar to previous studies in patients with primary hyperparathyroidism. Jonathan A. Sohn et al. conducted a study on 950 patients with primary hyperparathyroidism (parathyroid adenoma), of which 35% had renal insufficiency. The results of the study showed that although the reduction rates of Io-PTH in the group of patients with renal insufficiency were lower, but by changing the endpoint for Io-PTH evaluation for surgical success from 10 to 15 min, the diagnostic accuracy of this technique will increase from 89 to 95%. Hence, io-PTH measurement can also be used in patients with renal insufficiency. If the drop in serum PTH is more than 60% of baseline at 10 min, complete surgery can be ensured, and no further tissue exploration is required.

This procedure is especially useful in people who have multiple cervical explorations due to previous surgery or when three glands are found. However, in a study by Klaus Kaczirek et al. [35] on 35 SHPT patients who underwent bilateral cervical exploration and parathyroidectomy, io-PTH measurement was ineffective in determining the adequacy or inadequacy of parathyroid resection. Weber et al. also concluded that although the io-PTH method could guide diagnosing complete parathyroid resection, its diagnostic criteria should be selected more rigorously. Their study determined a reduction of more than 90% of io-PTH from baseline as a decision indicator [36].

**Fig. 2:**
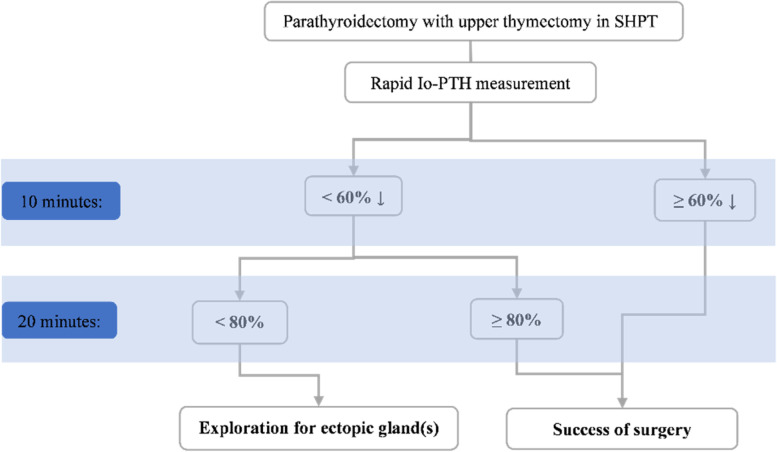
Effectiveness of reduction in rapid Intraoperative parathyroid hormone (Io-PTH) levels in managing secondary hyperparathyroidism (SHPT) patients undergoing parathyroidectomy with upper thymectomy

Based on the results of our study, we propose the following algorithm presented in Fig. 2 for the management of SHPT patients based on rapid Io-PTH measurements.

Based on our algorithm, a 60% or more reduction in PTH Rapid at 10 min after parathyroidectomy with upper thymectomy has an accuracy of 94.4% and a positive predictive value of 100%. Thus, if the PTH level does not decrease by more than 60% at 10 min or more than 80% at 20 min, accurate exploration should be performed again. If the remaining glands have not yet been identified, postoperative localization studies should be performed. On the other hand, if the rate of PTH reduction during surgery at 10 min is more than 60%, the surgery is considered successful, and the adequacy of treatment is considered. If the PTH decreases by less than 60% at 10 min, use the 20-min sample index after excision to make a decision. If the 20-min sample is less than 80%, tissue exploration is continued with the aim of finding the ectopic parathyroid gland.

Among the limitations of our study is the small sample size. Further multicentral studies would be beneficial in supporting the role of rapid Io-PTH in the management of SHPT surgery. Another limitation is that only two out of 36 patients in our study underwent total parathyroidectomy. Although our experience and other studies have reported no significant difference regarding the long-term effect of the method of parathyroidectomy on the PTH levels [23], further studies and explorations in this regard are warranted.

## Conclusion

We successfully managed SHPT in all our patients while also demonstrating the effectiveness of rapid Io-PTH in managing our patients. Based on the results of this study, a 60% or more reduction in PTH Rapid at 10 min after parathyroidectomy has an accuracy of 94.4% and a positive predictive value of 100%. Thus, if the PTH level does not decrease by more than 60% at 10 min or more than 80% at 20 min, tissue exploration is continued with the aim of finding the ectopic parathyroid gland.

## Data Availability

All data regarding this study has been reported in the manuscript. Please contact the corresponding author if interested in further information.
